# Identification of hsa_circRNA_100632 as a novel molecular biomarker for fulminant type 1 diabetes

**DOI:** 10.3389/fimmu.2023.1075970

**Published:** 2023-02-23

**Authors:** Wenfeng Yin, Shuoming Luo, Junlin Qiu, Zilin Xiao, Ziwei Zhang, Zhiguo Xie, Xia Li, Zhiguang Zhou

**Affiliations:** National Clinical Research Center for Metabolic Diseases, Key Laboratory of Diabetes Immunology (Central South University), Ministry of Education, and Department of Metabolism and Endocrinology, The Second Xiangya Hospital of Central South University, Changsha, Hunan, China

**Keywords:** circular RNAs, fulminant type 1 diabetes, biomarkers, diagnostic evaluation, immune regulation

## Abstract

**Objective:**

Circular RNAs (circRNAs) are associated with diabetes, but their role in fulminant type 1 diabetes (FT1D) is unclear. Thus, we characterized the role of circRNAs in FT1D.

**Research design and methods:**

CircRNA expression profiles were detected in peripheral blood mononuclear cells (PBMCs) of five FT1D patients and five controls using a circRNA microarray. An independent cohort comprised of 40 FT1D cases, 75 type 1 diabetes (T1D) cases, and 115 controls was used to verify the circRNAs using quantitative real-time polymerase chain reaction (qRT-PCR). Spearman’s correlation analysis and receiver operating characteristic (ROC) curve analysis were performed to determine the clinical diagnostic capability of circRNAs. Bioinformatics was used to identify potential biological functions and circRNA–miRNA–mRNA interactions.

**Results:**

There were 13 upregulated and 13 downregulated circRNAs in PBMCs of patients with FT1D. Five circRNAs were further verified in a second cohort. Hsa_circRNA_100632 was significantly upregulated in the FT1D and T1D groups. Hsa_circRNA_100632 was differentiated between patients with FT1D and controls [area under the curve (AUC) 0.846; 95% CI 0.776–0.916; *P*<0.0001] as well as between patients with FT1D and patients with T1D (AUC 0.726; 95% CI 0.633–0.820; *P*<0.0001). Bioinformatics analysis showed that hsa_circRNA_100632 may be involved in 47 circRNA–miRNA–mRNA signaling pathways associated with diabetes.

**Conclusions:**

CircRNAs were aberrantly expressed in PBMCs of patients with FT1D, and hsa_circRNA_100632 may be a diagnostic marker of FT1D.

## 1 Introduction

Fulminant type 1 diabetes (FT1D) is a novel subtype of type 1 diabetes (T1D) first identified by Imagawa et al. in 2000 (1, 2). FT1D is characterized by aggressive disease onset, dramatic loss of C-peptide concentration, nearly normal levels of hemoglobin A1c (HbA1c) at onset, and diabetic ketoacidosis (DKA) (3, 4). Failure to accurately diagnose FT1D can result in death (5, 6). Diagnosis of FT1D requires measurement of plasma glucose (PG) at onset, measurement of HbA1c, and onset of clinical symptoms of DKA within 1 week after onset. Then, fasting C-peptide (FCP) and 2 h postprandial C-peptide (PCP) must be measured after complete remission of ketoacidosis, which typically occurs within 1 or 2 weeks, to confirm the diagnosis (7, 8). These criteria make the diagnosis of FT1D difficult and complex, preventing precise intervention and worsening prognosis. Therefore, it is of great clinical significance to explore markers for identification of FT1D to allow for simple or early diagnosis and effective intervention.

Circular RNAs (circRNAs), a new class of non-coding RNAs, are characterized by a closed-loop structure, resistance to degradation, and excellent stability (9, 10). Several studies have shown that circRNAs are involved in many physiological and pathological disease processes, and circRNAs can serve as diagnostic and prognostic molecular biomarkers (10, 11). Our previous study showed that several circRNAs are differentially expressed in the peripheral blood of T1D patients and may contribute to T1D disease progression through interactions with miRNA and circRNA-derived peptides (12). Other studies have shown that circRNAs promote the onset and progression of T1D (13, 14). However, the expression profiles of circRNAs and the value of circRNAs as biomarkers of FT1D have not been characterized.

In the present study, we investigated the circRNA expression profiles in peripheral blood mononuclear cells (PBMCs) from patients with FT1D using microarray data, analyzed their biological functions using bioinformatics, and validated their expression levels using quantitative real-time polymerase chain reaction (qRT-PCR). As a result, a circRNA that can be used for diagnosis and differential diagnosis of FT1D was identified using a receiver operating characteristic (ROC) curve. The present study demonstrated that circRNAs have potential to aid in the diagnosis and treatment of patients with FT1D.

## 2 Research design and methods

### 2.1 Participants

A total of 240 individuals were placed into two cohorts, and **Table 1** shows the clinical and demographic data of these individuals. We selected five patients with new onset FT1D (median diabetes duration one month) and five age- and sex-matched control individuals for analysis in the first cohort. In the second cohort, 115 patients with FT1D (N = 40) or T1D (N = 75) and 115 controls were recruited to validate differentially expressed circRNAs identified in the first cohort.

**Table 1:** Demographic and clinical characteristics of study participants.

Variable	Control (N = 120)	Fulminant type 1 diabetes (N = 45)	Type 1 diabetes (N = 75)	*P* value
Age (years)	33.00 (26.25–41.75)	31.00 (26.50–44.00)	31.00 (22.0–39.00)	NS
Sex (male/female)	53/67	22/23	32/43	NS
BMI (kg/m^2^)	22.00 ± 2.68	21.23 ± 2.49	21.24 ± 2.66	NS
TG (mmol/L)	1.03 (0.69–1.59)	0.72 (0.59–1.03)	0.64 (0.52–0.88)	< 0.001
TC (mmol/L)	4.65 (4.13–5.17)	4.33 (3.91–4.71)	4.27 (3.66–4.84)	NS
LDL-C (mmol/L)	2.70 (2.29–3.31)	2.32 (1.91–2.90)	2.33 (1.73–3.00)	< 0.01
HDL-C (mmol/L)	1.38 (1.15–1.67)	1.52 (1.29–1.82)	1.57 (1.26–1.84)	< 0.05
FPG (mmol/L)	4.74 ± 0.42	9.83 ± 4.15	7.94 ± 3.58	< 0.001
FCP (ng/mL)	—	0.05 (0.05–0.08)	0.12 (0.05–0.44)	< 0.0001
HbA1c (%)	5.40 (5.20–5.60)	7.20 (6.40–8.08)	7.20 (6.30–8.25)	< 0.001
HbA1c (mmol/mol)	36 (33–38)	55 (46–65)	55 (45–67)	< 0.001

BMI, body mass index; TG, triglyceride; TC, total cholesterol; LDL-C, low density lipoprotein-cholesterol; HDL-C, high density lipoprotein-cholesterol; FPG, fasting plasma glucose; FCP, fasting C-peptide; HbA1c, hemoglobin A1c; NS, not significant.

Patients with diabetes met the diagnostic criteria of the American Diabetes Association (15). Individuals with T1D were recruited based on the following criteria: (1) acute onset of ketosis or ketoacidosis requiring insulin replacement therapy; (2) at least six months of continuous insulin dependence after diagnosis; and (3) positive glutamic acid decarboxylase antibodies (GADAs). Diagnosis of FT1D was performed according to the criteria of the Committee of the Japan Diabetes Society in 2012 (16). The inclusion criteria for FT1D were as follows (1): occurrence of diabetic ketosis or ketoacidosis within a week after onset of hyperglycemic symptoms; (2) initial PG level > 16.0 mmol/L and HbA1c < 8.7% (72 mmol/mol) at onset; and (3) serum FCP < 100 pmol/L (0.3 ng/ml) and PCP < 170 pmol/L (0.5 ng/ml) at disease onset. Controls had fasting plasma glucose (FPG) < 5.6 mmol/L and HbA1c < 6.1%. The exclusion criteria for control subjects were infectious diseases, pregnancy, malignant disease, or a family history of diabetes.

All data were collected from patients from the Second Xiangya Hospital of Central South University (Changsha, Hunan, China). The study was approved by the Ethical Committee of the Second Xiangya Hospital of Central South University (LYF 2021100), and all subjects provided written informed consent prior to inclusion in the study.

### 2.2 Data collection and clinical measurements

Sex, age, body height, and weight were recorded for all subjects. Fasting venous blood samples were tested for triglycerides (TG), total cholesterol (TC), high density lipoprotein-cholesterol (HDL-C), low density lipoprotein-cholesterol (LDL-C), FPG, and HbA1c using standard procedures in the hospital clinical laboratory. GADAs were detected as previously described (17).

### 2.3 Peripheral blood mononuclear cell collection

Fasting peripheral blood was collected from patients and controls into EDTA blood tubes, and PBMCs were isolated using density gradient centrifugation. Ficoll-Paque PLUS (GE Healthcare, USA) was added to the blood samples with an equal amount of phosphate buffered saline (PBS; Invitrogen, USA). Finally, PBMCs were stored in TRIzol reagent (Invitrogen, USA) at –80 °C.

### 2.4 Total RNA extraction and quality control

Total RNA was extracted from PBMCs of each participant using TRIzol reagent (Invitrogen, USA) according to the manufacturer’s instructions. The RNA purity and concentration were determined using a NanoDrop 2000 instrument (Thermo Scientific, USA). The range of mean A260/A280 was 1.8 to 2.0.

### 2.5 CircRNA microarray expression profiling

Sample preparation and microarray hybridization were performed according to the manufacturer’s instructions (Arraystar Human circRNA Array V2.0, 8 ×15K). Total RNA was digested with Rnase R (Epicentre, USA) to remove of linear RNA and enrich circRNA. The enriched circRNAs were then amplified and transcribed into fluorescent cRNA according to the Arraystar Super RNA Labeling protocol (Arraystar, USA). Finally, the hybridized arrays were washed, fixed, and scanned using an Agilent Scanner G2505C. The details of the method have been previously described (12). Agilent Feature Extraction software (version 11.0.1.1) was used to analyze the acquired array images. Quantile normalization and subsequent data processing were performed using the limma package in R software. Significantly differentially expressed circRNAs between the FT1D group and control group were identified using volcano plot filtering. CircRNAs with absolute fold differences ≥ 1.5 and *P* < 0.05 were considered significantly differentially expressed. Hierarchical clustering was performed to show the distinguishable circRNA expression pattern among samples.

### 2.6 GO and KEGG pathway enrichment analyses

OmicShare tools was used to perform Gene Ontology (GO) and Kyoto Encyclopedia Genes and Genomes (KEGG) analyses. GO analysis was conducted based on the parent genes of circRNAs to investigate the properties of aberrantly expressed circRNAs. Pathways were analyzed based on differentially expressed circRNAs using KEGG analysis.

### 2.7 QRT-PCR assay

One microgram of total RNA was converted into cDNA using a GoScript™ Reverse Transcription System (Promega, USA) according to the manufacturer’s instructions. Target gene expression levels were determined using a SYBR Green kit (Promega, USA) in 10-μl samples using an ABI PRISM Step One Sequence Detection System (Applied Biosystems, USA). The thermocycler conditions were as follows: 95°C for 10 min followed by 45 cycles of 95°C for 15 s and 60°C for 1 min. Internal standard was β-actin, and all reactions were performed in triplicate. The designed and optimized primers for circRNA transcripts are shown in **Supplemental Table S1**.

### 2.8 CircRNA–miRNA–mRNA network analysis

CircRNA can sponge miRNA, and mRNA can be targeted by miRNA, which may play critical roles in disease onset and progression. To elucidate the relationship between circRNAs and miRNAs, TargetScan and miRanda prediction software were used to predict the interactions of circRNAs–miRNAs–mRNAs. The circRNA–miRNA–mRNA network was constructed using the Cytoscape bioinformatics software.

### 2.9 Statistical analysis

Data are presented as the mean ± SD (standard deviation) or as the median (25th–75th percentile). The 2^-ΔΔCT^ method was used to calculate the relative expression levels of selected circRNAs analyzed using qRT-PCR. Normality of the data was checked using the Shapiro–Wilk test. One-way analysis of variance (ANOVA) was performed to compare groups if the data were normally distributed, and a rank test (Mann–Whitney U test or Kruskal–Wallis H test) was used if the data were not normally distributed. The Chi-square test was used to analyze categorical data among groups. Generalized linear regression model analysis was used to identify the associations between circRNA expression levels and FT1D after adjusting the confounders. Spearman’s correlation coefficient was used to evaluate the relationship between circRNAs and clinical characteristics of FT1D. A ROC curve was used to assess the diagnostic and differential diagnostic performance of circRNA for FT1D. Statistical analyses were performed using SPSS 21.0 (IBM SPSS, Inc., Chicago, IL, USA) and GraphPad Prism 5.01 (GraphPad Software, San Diego, CA, USA). *P* < 0.05 was considered statistically significant.

## 3 Results

### 3.1 Identification of aberrantly expressed circRNAs in PBMCs of patients with FT1D

**Figure 1:**
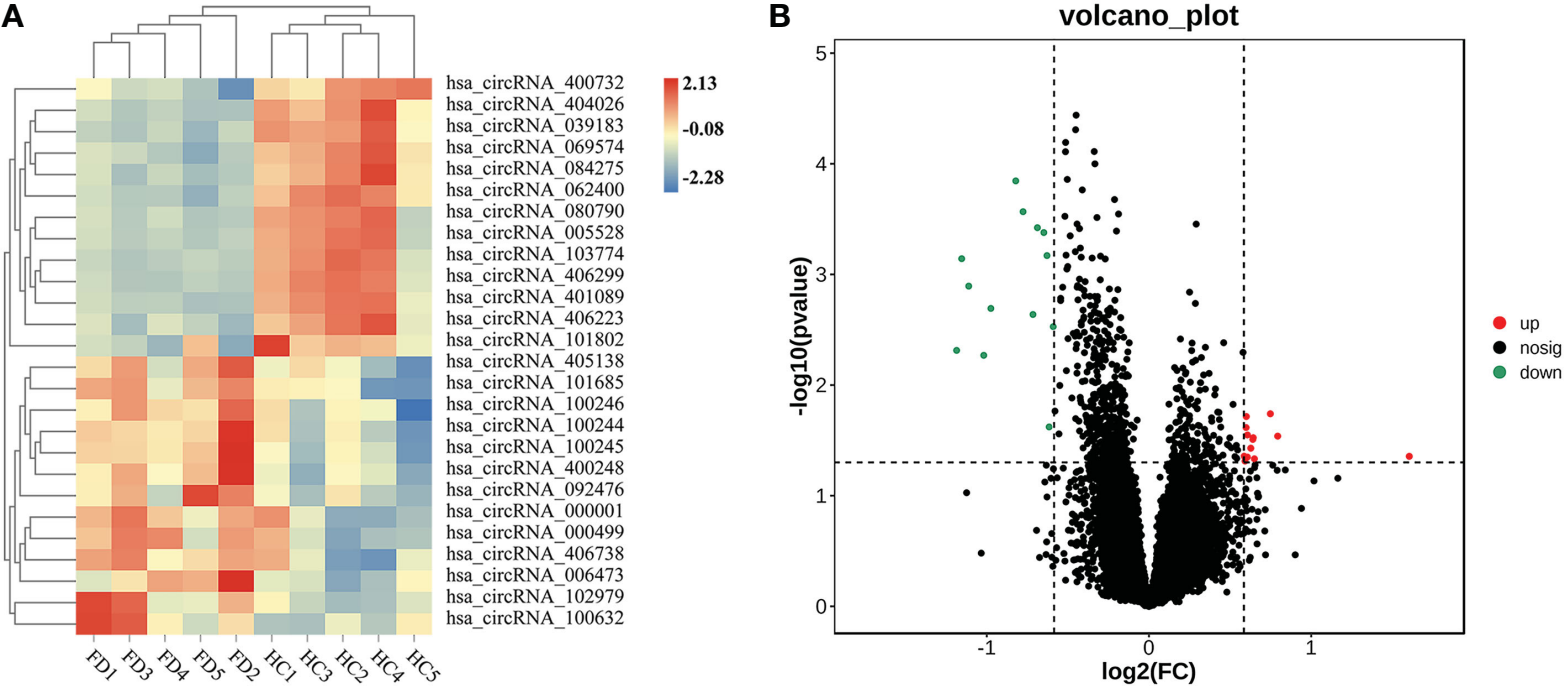
Expression profiles of significantly altered circRNAs in peripheral blood mononuclear cells (PBMCs) of 5 patients with fulminant type 1 diabetes and 5 controls. **(A)** Heatmap of 26 differentially expressed circRNAs in PBMCs of patients with fulminant type 1 diabetes and controls. **(B)** Twenty-six significantly upregulated and downregulated circRNAs according to volcano diagram analysis (absolute fold changes ≥ 1.5, *P* < 0.05).

We detected 13,563 circRNAs in PBMCs from patients with FT1D. The classification of circRNAs according to expression level was performed by hierarchical clustering (**Figure 1A**). In the volcano plots (**Figure 1B**), separately expressed circRNAs were grouped by absolute fold changes ≥ 1.5 and *P* < 0.05, resulting in 26 differentially expressed circRNAs in patients with respect to controls, of which 13 were upregulated and 13 were downregulated (**Table 2**).

**Table 2:** The detailed information of the differentially expressed circRNAs in fulminant type 1 diabetes.

circRNA name	chrom	GeneSymbol	*P* value	FC	regulation
hsa_circRNA_405138	chr13	EPSTI1	0.0464131	1.5685806	up
hsa_circRNA_092476	chr19	VN1R1	0.0439919	1.5016777	up
hsa_circRNA_102979	chr20	RBCK1	0.028246	1.5227779	up
hsa_circRNA_000001	chr1	None	0.0486577	1.5073131	up
hsa_circRNA_006473	chr4	ARHGAP10	0.0242112	1.5142405	up
hsa_circRNA_101685	chr16	ABCA3	0.0299112	1.5608572	up
hsa_circRNA_000499	chr13	RBM26	0.0289557	1.7330466	up
hsa_circRNA_100246	chr1	FGGY	0.0448004	1.5225467	up
hsa_circRNA_100244	chr1	FGGY	0.0309526	1.5584043	up
hsa_circRNA_406738	chr6	CD83	0.0371987	1.5448885	up
hsa_circRNA_100245	chr1	FGGY	0.0181813	1.6795064	up
hsa_circRNA_100632	chr10	SAMD8	0.0440794	3.0407460	up
hsa_circRNA_400248	chr1	FGGY	0.0192492	1.5155139	up
hsa_circRNA_400732	chr11	QSER1	0.0023017	1.6418904	down
hsa_circRNA_404026	chr8	SMIM19	0.0004186	1.5672501	down
hsa_circRNA_039183	chr16	ZNF267	0.0002711	1.7126636	down
hsa_circRNA_080790	chr7	YWHAG	0.0053845	2.0259209	down
hsa_circRNA_005528	chr2	ANAPC1P1	0.0048599	2.2748517	down
hsa_circRNA_406223	chr3	SEC13	0.0029659	1.5057239	down
hsa_circRNA_101802	chr16	GPT2	0.0238991	1.5321563	down
hsa_circRNA_069574	chr4	UCHL1	0.0003781	1.6109350	down
hsa_circRNA_103774	chr4	WWC2	0.0020291	1.9652714	down
hsa_circRNA_406299	chr3	TMF1	0.0012755	2.1603710	down
hsa_circRNA_401089	chr12	VPS33A	0.000721	2.2261375	down
hsa_circRNA_062400	chr22	CRKL	0.0001428	1.7669508	down
hsa_circRNA_084275	chr8	PRKDC	0.0006759	1.5463376	down

FC, absolute fold changes.

### 3.2 Bioinformatics analysis of differentially expressed circRNAs

**Figure 2:**
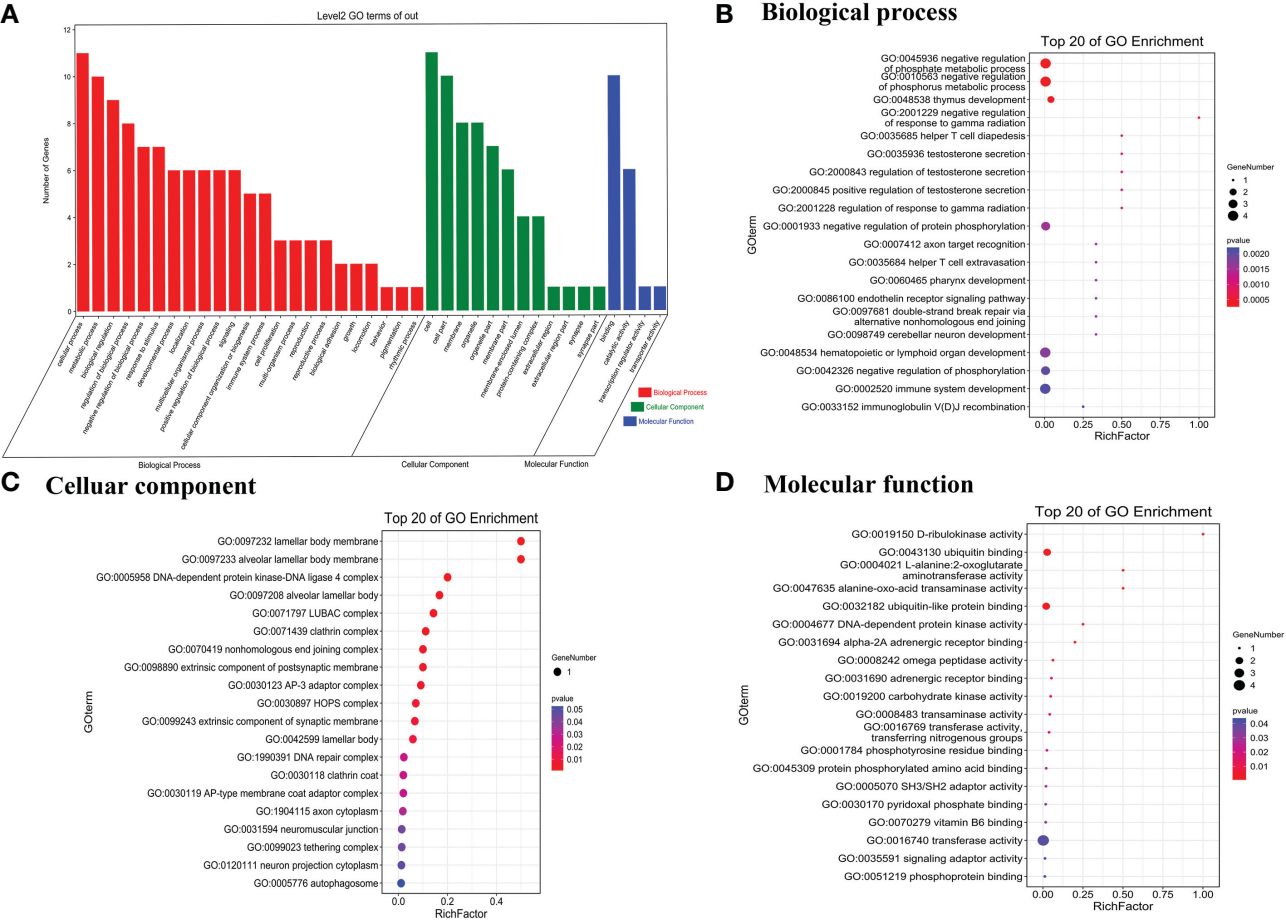
Gene Ontology terms of parent genes of differentially expressed circRNAs. **(A)** Gene Ontology categories of significantly differentially expressed circRNAs. **(B)** Biological process, **(C)** cellular component, and **(D)** molecular function of the top 20 genes in GO enrichment analysis.

To predict the potential biological functions of the 26 differentially expressed circRNAs, we used their parent genes for GO enrichment and KEGG pathway analyses. The GO terms for parent genes of differentially expressed circRNAs were classified and summarized according to biological process (BP), cellular component (CC), and molecular function (MF). The terms with the most genes in BP, CC, and MF were cellular process, binding, and cell, respectively (**Figure 2A**). For BP, the term with the highest enrichment score was negative regulation of phosphate metabolic process (**Figure 2B**), and the term with the highest enrichment score for CC was lamellar body membrane and alveolar lamellar body membrane (**Figure 2C**). For MF, the term with the highest enrichment score was D-ribulokinase activity (**Figure 2D**).

**Figure 3:**
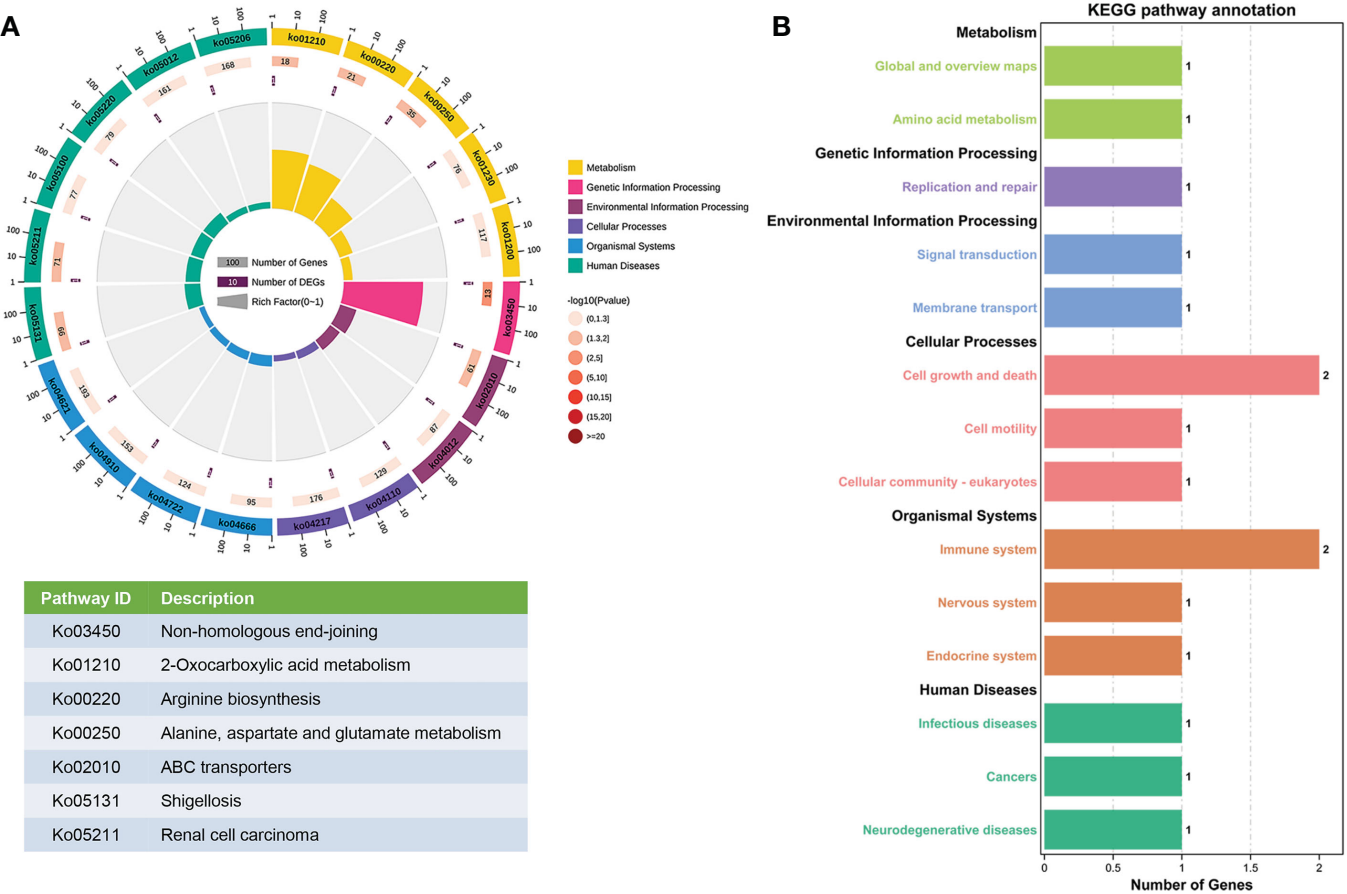
KEGG pathway gene enrichment analysis for differentially expressed circRNAs. **(A)** Enriched circle map of the transcripts of 26 significantly differentially expressed circRNAs. The first circle indicates the top 20 KEGG pathways. The second circle indicates the gene number for the specified signaling pathway. The third circle indicates the total number of prospective genes. The fourth circle indicates the enrichment factor of each KEGG pathway. **(B)** KEGG pathway enrichment analysis of genes that produced up- and downregulated circRNAs.

The enriched pathways of the parent genes of the 26 differentially expressed circRNAs were analyzed using KEGG pathway annotation. The non-homologous end-joining and 2-oxocarboxylic acid metabolism terms had the highest correlations with the enrichment scores (**Figure 3A**). Furthermore, KEGG pathway annotation showed that most genes were involved in cell growth and death as well as the immune system (**Figure 3B**).

### 3.3 Validation of differentially expressed circRNAs

**Figure 4:**
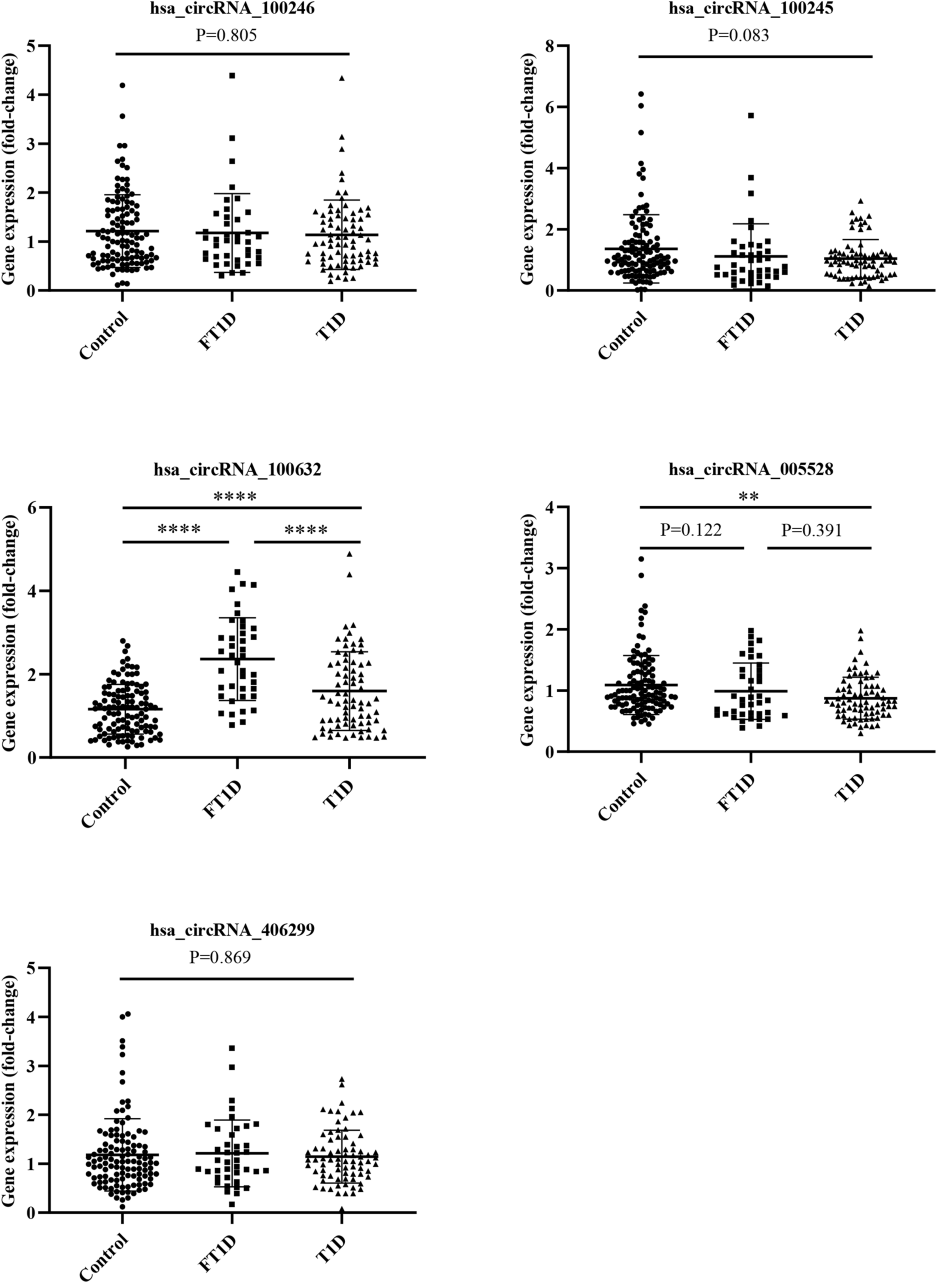
QRT-PCR analysis of five differentially expressed circRNAs among patients with fulminant type 1 diabetes, patients with type 1 diabetes, and controls. (fulminant type 1 diabetes, N = 40; type 1 diabetes, N = 75; controls N = 115). ^**^ *P* < 0.01 and ^****^ *P* < 0.0001.

To further validate the microarray data, five differentially expressed circRNAs, including three upregulated circRNAs (hsa_circRNA_100246, hsa_circRNA_100245, and hsa_circRNA_100632) and two downregulated circRNAs (hsa_circRNA_005528 and hsa_circRNA_406299), were selected for qRT-PCR verification. The following four inclusion criteria were used: (1) circRNA with a high differential expression fold; (2) circRNA length between 200 bp and 1000 bp; (3) circRNA average raw intensity > 100; and (4) exonic-circRNAs. As shown in **Figure 4**, patients with FT1D and T1D had significantly increased expression of hsa_circRNA_100632 (*P*<0.0001) and significantly decreased expression of hsa_circRNA_005528 (*P*<0.01) compared to controls. Moreover, patients with FT1D showed higher expression of hsa_circRNA_100632 than patients with T1D (*P*<0.0001) and hsa_circRNA_100632 has a significant difference in FT1D and controls (*P*<0.0001) regardless of before or after adjusting for age (**Table S2**).

### 3.4 Association of significantly differentially expressed circRNAs with clinical parameters

To analyze whether significantly differentially expressed circRNAs are associated with clinical parameters, Spearman’s correlation analysis was performed in the FT1D group, the T1D group, the control group, and for all subjects. In patients with T1D, the expression levels of hsa_circRNA_100632 were positively correlated with FCP (*P*<0.05), and the expression levels of hsa_circRNA_005528 were positively associated with sex (*P*<0.01). In all subjects, hsa_circRNA_100632 was negatively correlated with age (*P*<0.05), BMI (*P*<0.01), TG (*P*<0.05), TC (*P*<0.01), and LDL-C (*P*<0.01), but hsa_circRNA_100632 was positively associated with FPG (*P*<0.01) and HbA1c (*P*<0.01). In addition, hsa_circRNA_005528 was negatively correlated with HbA1c in all subjects (*P*<0.01) (**Table 3**).

**Table 3:** Spearman’s correlation between the expression levels of circRNAs and anthropometric and metabolic variables.

	Age	Sex	BMI	TG	TC	LDL-C	HDL-C	FPG	FCP	HbA1c
Total subjects										
hsa_circRNA_100632	–0.169*	0.019	–0.175**	–0.195*	–0.205**	–0.241**	0.045	0.200**	0.042	0.329**
hsa_circRNA_005528	–0.097	0.059	0.046	–0.021	0.031	0.099	–0.146	–0.073	0.035	–0.200**
Control group										
hsa_circRNA_100632	–0.251**	0.063	–0.159	–0.002	–0.117	–0.178	–0.186	0.046	–	–0.185
hsa_circRNA_005528	–0.094	0.004	–0.035	–0.017	0.005	–0.009	–0.049	0.039	–	–0.183
Fulminant type 1 diabetes group										
hsa_circRNA_100632	–0.196	–0.035	0.136	0.028	0.125	–0.023	0.120	–0.126	–0.084	0.170
hsa_circRNA_005528	–0.302	–0.165	–0.158	–0.084	0.170	0.275	–0.154	–0.099	0.245	0.065
Type 1 diabetes group										
hsa_circRNA_100632	–0.142	–0.027	–0.196	–0.220	–0.235	–0.205	0.006	–0.067	0.263*	0.172
hsa_circRNA_005528	–0.098	0.305**	0.160	–0.097	–0.199	–0.074	–0.194	–0.057	–0.030	–0.048

BMI, body mass index; TG, triglyceride; TC, total cholesterol; LDL-C, low density lipoprotein-cholesterol; HDL-C, high density lipoprotein-cholesterol; FPG, fasting plasma glucose; FCP, fasting C-peptide; HbA1c, hemoglobin A1c; ^*^P < 0.05, ^**^P < 0.01

### 3.5 Diagnostic value of hsa_circRNA_100632 as a biomarker of FT1D

**Figure 5:**
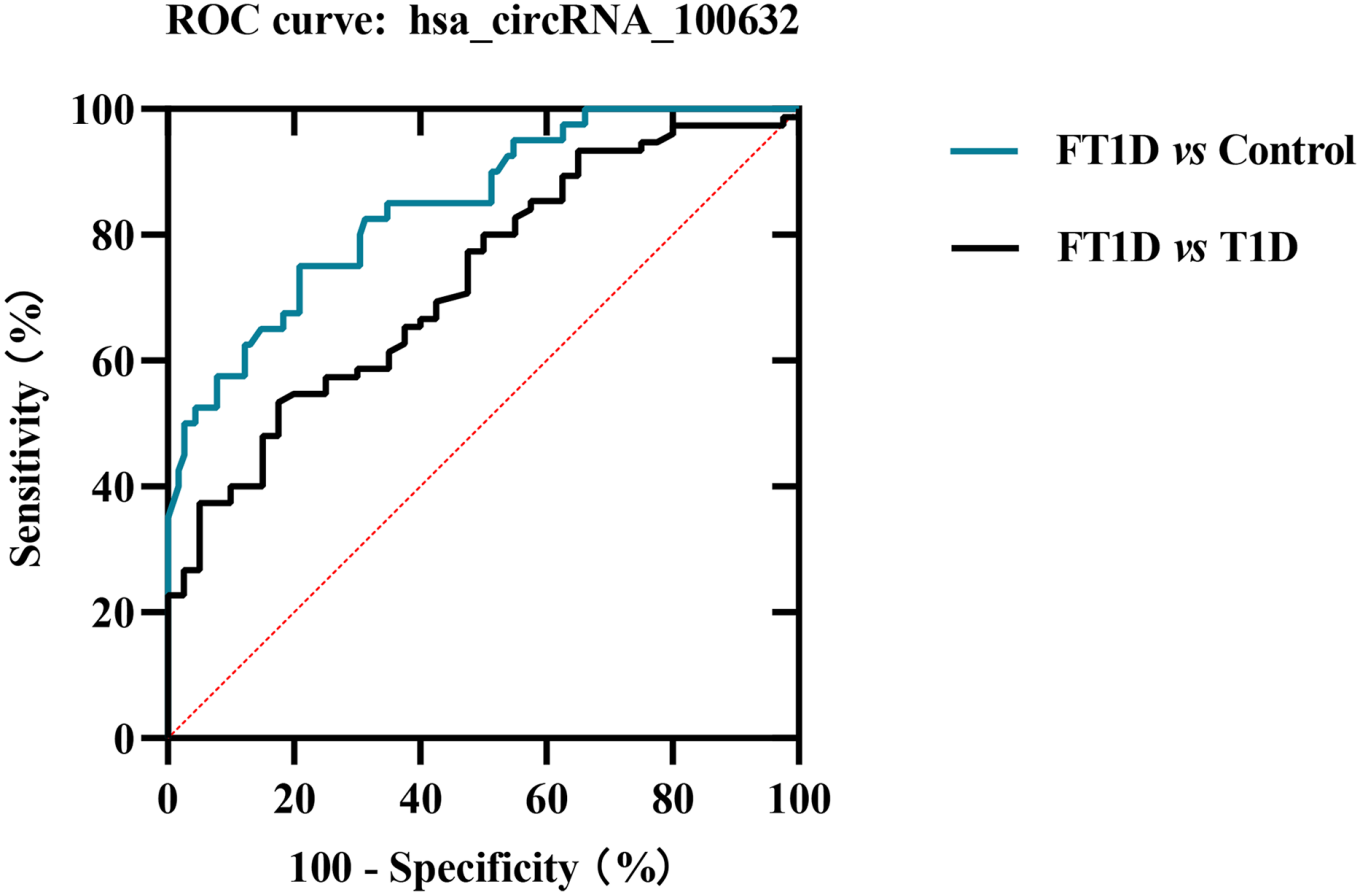
Receiver operating characteristic (ROC) plot of hsa_circRNA_100632 in fulminant type 1 diabetes. The blue line indicates the ROC curve for hsa_circRNA_100632 distinguishing fulminant type 1 diabetes from controls, and the black line represents the ROC curve for hsa_circRNA_100632 distinguishing fulminant type 1 diabetes from type 1 diabetes.

To further evaluate whether has_circRNA_100632 has molecular diagnostic value for FT1D, we generated a ROC curve to evaluate its sensitivity and specificity. As shown in **Figure 5**, the area under the curve (AUC) for hsa_circRNA_100632 was 0.846 (95% CI 0.776–0.916) with a sensitivity of 75.0% and specificity of 79.1% between patients with FT1D and controls (*P*<0.0001). Furthermore, the AUC of hsa_circRNA_100632 was 0.726 (95% CI 0.633–0.820) with a sensitivity of 82.5% and specificity of 58.8% between patients with FT1D and patients with T1D (*P*<0.0001).

### 3.6 Construction of circRNA–miRNA–mRNA interaction network

**Figure 6:**
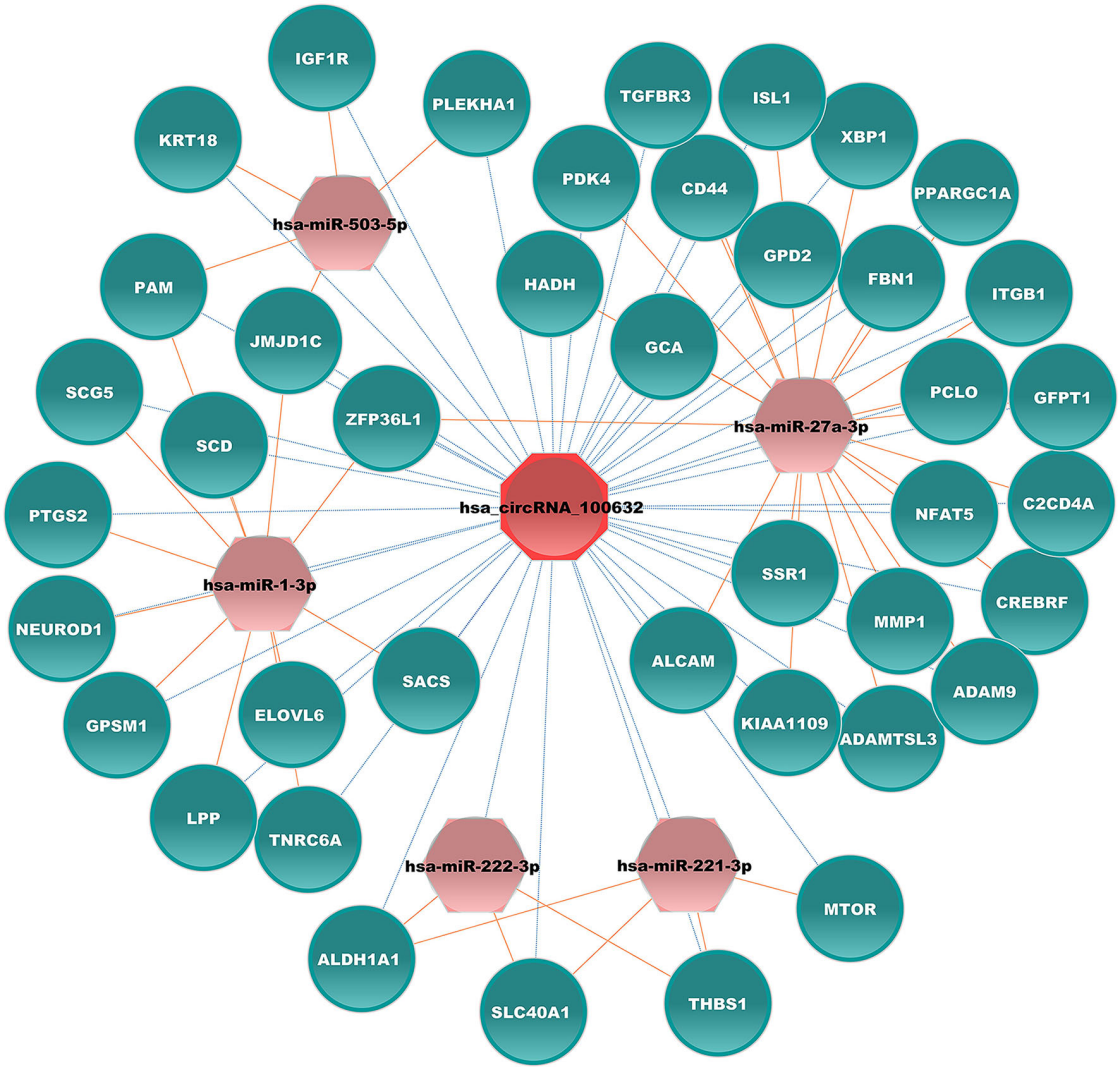
Hsa_circRNA_100632–miRNA–mRNA network. Blue ellipses, pink hexagons, and red octagons represent mRNAs related to diabetes, diabetes-associated miRNAs, and hsa_circRNA_100632, respectively. The orange solid lines indicate the relationship between miRNAs and mRNAs, and the dotted lines represent the relationship between circRNAs and miRNAs.

CircRNAs can function as competing endogenous RNA that can sponge corresponding miRNAs and affect mRNA expression in various diseases, including diabetes. Hsa_circRNA_100632 was predicted by circBank, a comprehensive database of human circRNA, to sponge 70 miRNAs. Five miRNAs related to diabetes were selected (hsa-miR-1-3p, hsa-miR-27a-3p, hsa-miR-221-3p, hsa-miR-222-3p, and hsa-miR-503-5p) for miRNA–mRNA prediction. According to TargetScan and miRanda software, these 5 miRNAs may bind to 333 mRNAs. We next selected 41 mRNAs related to diabetes to form a specific circRNA–miRNA–mRNA network related to diabetes. The results showed that hsa_circRNA_100632 may be involved in 47 circRNA–miRNA–mRNA signaling pathways associated with diabetes (**Figure 6**). For example, hsa_circRNA_100632 sponges hsa-miR-221-3p and hsa-miR-222-3p, resulting in modulation of the thrombospondin 1 (THBS1), solute carrier family 40 member 1 (SLC40A1), and aldehyde dehydrogenase 1 family, member A1 (ALDH1A1) target genes.

## 4 Discussion

In the present study, we profiled circRNA expression in PBMCs of patients with FT1D. We verified that hsa_circRNA_100632 was differentially upregulated and that hsa_circRNA_005528 was differentially downregulated among patients with FT1D, patients with T1D, and controls. We found that hsa_circRNA_100632 levels were associated with islet β-cell destruction in patients with T1D. Previous studies have suggested that circRNAs may play a role in T1D or type 2 diabetes (T2D) through regulation of islet β-cell dysfunction (18, 19). CircGlis3, a β-cell-derived exosomal circRNA, has been shown to play a role in lipotoxicity-induced β-cell disorder and development of diabetes through suppression of insulin secretion and cell proliferation (20). In the present study, we identified hsa_circRNA_100632 as an important circRNA in FT1D. Has_circRNA_100632 is derived from sterile alpha motif domain containing 8 (SAMD8), which is a ceramide phosphoethanolamine synthase in the endoplasmic reticulum. This circRNA may act as a ceramide sensor, and its N-terminal SAM structural domain controls endoplasmic reticulum ceramide levels and inhibits ceramide-induced mitochondrial apoptosis (21). High glucose can lead to mitochondrial dysfunction through oxidative stress (22). We hypothesized that hsa_circRNA_100632 may promote progression of T1D through induction of β-cell failure. Future studies that include functional experiments using immune cells and animal models are needed.

The present study indicated that hsa_circRNA_100632 in PBMCs may be a diagnostic marker of FT1D. Increasing evidence has indicated that circRNAs may be diagnostic indicators of T1D and T2D (23). Hsa_circ_0054633, a diagnostic biomarker of pre-diabetes and T2D, shows diagnostic capability with AUC values of 0.751 and 0.793, respectively (24). In addition, hsa_circ_0063425 and hsa_circ_0056891 may be valuable markers for early detection of T2D (25). A recent study has suggested that the expression level of hsa_circ_0060450 is upregulated in patients with T1D and may represent a novel therapeutic target for T1D (14). Furthermore, our previous study found that hsa_circ_0072697 is involved in 50 signaling pathways related to diabetes, indicating that it may be a diagnostic indicator of T1D (12). However, the role of circRNA in FT1D has not been characterized. The present study is the first to indicate that circRNAs may play a key role in FT1D, suggesting that circRNAs may be valuable diagnostic and differential diagnostic markers for FT1D. ROC analysis showed that hsa_circRNA_100632 had a sensitivity of 75.0% and specificity of 79.1% for distinguishing between patients with FT1D and control subjects (AUC = 0.846). Moreover, hsa_circRNA_100632 distinguished between FT1D and T1D with 82.5% sensitivity and 58.8% specificity (AUC = 0.726). Several diagnostic markers have been previously explored in smaller cohorts, and models have been built from data derived from multiple clinical studies (5, 26–28). For example, the glycated albumin (GA)/HbA1c ratio has been verified as a sensitive marker for glucose excursion of FT1D in a cohort comprised of 56 outpatients (27). The fulminant index shows an ability to identify FT1D based on DKA (5). In addition, the serum 1,5-anhydroglucitol/GA index has been shown to be a suitable indicator for early differential diagnosis between FT1D and T1D when HbA1c < 8.7% with an optimal cut-off point of 0.3 (28). In contrast, the present study focused on the expression levels of hsa_circRNA_100632, which may simplify and reduce the cost associated with diagnosis of FT1D.

In addition, KEGG analysis showed that differentially expressed circRNAs may be associated with cell growth and death as well as the immune system. FT1D has a rapid onset, severe symptoms, and a high mortality rate (29, 30). Initially, FT1D was considered related to non-autoimmune pathogenesis due to the absence of islet autoantibodies, such as GADAs (1). Over the past 20 years, FT1D has gained increasing attention. Studies have shown that immunity plays a critical role in the initiation and progression of FT1D (29, 31). Cellular and humoral immunity may play an important role in FT1D (29). Viral infection and drug induction may lead to β-cell loss in FT1D by triggering an immune response (32, 33). The present results indicated that circRNAs were abnormally expressed in peripheral immune cells in FT1D, and bioinformatics analysis indicated that circRNAs may be involved in the immune response. Furthermore, the present results showed that hsa_circRNA_100632 may be involved in 47 diabetes-associated circRNA–miRNA–mRNA interaction signaling pathways. In the hsa_circRNA_100632 network, hsa_circRNA_100632 may sponge hsa-miR-27a-3p and modulate the nuclear factor of activated T cells 5 (NFAT5) target gene. NFAT5 activates various immune cells, especially T lymphocytes (34). These findings suggest that hsa_circRNA_100632/hsa-miR-27a-3p/NFAT5 may play a key role in FT1D by regulating the immune response. However, it is not known whether the upregulated expression levels of hsa_circRNA_100632 are due to increased expression of specific leukocytes in PBMCs of FT1D patients or whether the increasing hsa_circRNA_100632 can affect specific leukocyte expression. Further studies are needed to verify this finding.

The major strength of the present work was that it included a large validation cohort considering the rarity of FT1D. Furthermore, the protocols for recruitment and examination of subjects were highly standardized. However, the present study had several limitations. The sample size of the discovery cohort was small and the multiple-testing correction was not performed in the discovery analysis, indicating that the relevance of hsa_circRNA_100632 to the pathogenesis of FT1D requires further immune cell and animal studies. In addition, the present study focused on FT1D in a Chinese population, and further studies are needed to determine if these results regarding hsa_circRNA_100632 can be generalized to other ethnic groups.

In summary, we characterized the circRNA transcriptome in PBMCs derived from patients with FT1D for the first time. We then predicted the biological functions of differentially expressed circRNAs using GO and KEGG pathway analyses. The results indicated that hsa_circRNA_100632 may be a molecular biomarker for FT1D. Longitudinal studies are needed to validate hsa_circRNA_100632 as a molecular biomarker of FT1D progression.

## Data availability statement

The original contributions presented in the study are publicly available. This data can be found here: https://ngdc.cncb.ac.cn/omix.(OMIX repository, accession number OMIX002351).

## Ethics statement

The studies involving human participants were reviewed and approved by the ethical committee of the Second Xiangya Hospital of Central South University. The patients/participants provided their written informed consent to participate in this study. Written informed consent was obtained from the individual(s) for the publication of any potentially identifiable images or data included in this article.

## Author contributions

WY contributed to the experiments and data analysis and wrote the first draft of the manuscript. SL and ZZho proposed the project, designed the study, provided critical suggestions, and revised the manuscript. JQ, ZXia, and ZZha collected the study data and samples. JQ, ZXie, and XL reviewed the manuscript and contributed to the discussion. All authors contributed to the article and approved the submitted version.
