# Supplementary material for: Identification of hsa_circRNA_100632 as a novel molecular biomarker for fulminant type 1 diabetes
Source: Front Immunol. 2023 Feb 23;14:1075970. doi: 10.3389/fimmu.2023.1075970 (PMC9996325; doi:10.3389/fimmu.2023.1075970)
Supplement: Supplementary file 1 [file Table_1.docx]

| **Table S1: List of primers used for quantitative real-time PCR** | | | |
| --- | --- | --- | --- |
| **Gene name** | **Two-way primer sequence** | **Annealing Temperature (℃)** | **Product length (bp)** |
| β-actin（H） | F:5' GTGGCCGAGGACTTTGATTG3'  R:5’ CCTGTAACAACGCATCTCATATT3’ | 60 | 73 |
| hsa_circRNA_100632 | F:5’ CTGTATTCTTGCTTCGCTGCTT 3’  R:5’ CTCCTCCGCTGCCTTTCC 3’ | 60 | 92 |
| hsa_circRNA_100246 | F:5’ ACAGTGTCCTCCAGTACGTC 3’  R:5’ TCCCTTGTACAACTTTCGTGC 3’ | 60 | 179 |
| hsa_circRNA_100245 | F:5’ AGCACAGTGTCCTCCAGTAC 3’  R:5’ ATTGCAGTTCCTCCACCGT 3’ | 60 | 182 |
| hsa_circRNA_005528 | F:5’ ACATGGCAGCTCTAAGGAAGA 3’  R:5’ AGGCTTCTGAGATGTGCTGT 3’ | 60 | 108 |
| hsa_circRNA_406299 | F:5’ CTCCTTGCTAACAAAATTCAGA 3’  R:5’ CCTAAGGTCCCCCACCTAT 3’ | 60 | 149 |

| **Table S2: Generalized linear regression model analysis of the relationship between circRNA and FT1D** | | |
| --- | --- | --- |
| **Variables** | **Adjusted for age** | |
|  | ***β* coefficient** | ***P*** |
| Age | 0.006 | 0.019 |
| hsa_circRNA_100632 | 0.306 | ＜0.0001 |
